# Network Pharmacology-Based Strategy to Reveal the Mechanism of Cassiae Semen against Cataracts

**DOI:** 10.1155/2022/5654120

**Published:** 2022-07-11

**Authors:** Ying Zhong, Ruo-fu Chen, You-fa Fang

**Affiliations:** ^1^Department of Ophthalmology, Shangyu People's Hospital of Shaoxing, Shaoxing, Zhejiang 312300, China; ^2^Department of Ophthalmology, Yongkang First People's Hospital, Yongkang, Zhejiang 321300, China

## Abstract

Cassiae semen (CS) is one of the most well-known herbs used in the treatment of cataracts in China. However, the potential mechanisms of its anticataract effects have not been fully explored. In this study, network pharmacology was used to investigate the potential mechanism underlying the actions of CS against cataracts, and molecular docking was performed to analyze the binding activity of proteins and compounds. qPCR was performed to detect the mRNA level of genes, and the cell apoptotic rate was measured using flow cytometry. We identified 13 active compounds from CS and 105 targets, as well as 238 cataract-related targets. PPI networks were constructed, and fifty key targets were obtained. These key targets were enriched in the regulation of transcription, apoptotic process, and signal transduction pathways. Molecular docking demonstrated that the compounds of CS exhibited good affinity to some critical targets. Furthermore, CS prevented the apoptosis of human lens epithelial cells induced by UVB lights by decreasing the gene expression of CASP3, ESR1, and TP53 and increasing the CRYAB gene expression. The present study attempted to explain the mechanisms for the effects of CS in the prevention and treatment of cataracts and provided an effective strategy to investigate active ingredients from natural medicines. Further studies are required to verify these findings via *in vivo* and *in vitro* experiments.

## 1. Introduction

Cataract is currently the main cause of visual impairment and blindness globally, accounting for 46 percent of blind people. Visual impairment leads to a series of difficulties in patients' daily life and social problems, which would contribute to an extensive economic burden on society [1]. Up to date, surgery is the main method for the treatment of cataracts. Nevertheless, in developing countries, owing to the limited access to surgery caused by a higher prevalence of blindness due to cataracts and lack medical resources [2], it is urgent to develop pharmacological strategies for the management of cataracts. Based on the mechanism of cataracts' formation, herbal, minerals, amino acids, and antioxidants were developed to treat cataracts. Meanwhile, there are other available approaches by inhibiting glycation, phase separation, matrix metalloproteinase, and modulating the TGF-*β* pathway [3].

Cassiae semen (CS), the seed of *Cassia obtusifolia* L. or *Cassia tora* L. of the family Leguminosae, was initially recorded in the earliest book of Chinese materia medica “Shennong Bencao Jing” and described for treating dizziness and headache, improving vision, and nourishing the liver [4]. Modern pharmacological studies reported the therapeutic potential of *Cassia tora* leaves in preventing cataracts [5, 6]. It has been revealed that anthraquinone compounds, including obtusin, emodin, and aloe emodin, are the main bioactive components in CS [7–9]. In addition, a recent study suggested that emodin could serve as a potential therapeutic agent for cataracts [10], and the antioxidant activity of active ingredients from CS has also been confirmed in many studies [11–13], which may be used as antioxidants for cataracts. However, although many studies confirmed that CS showed noticeable anticataract effects, the underlying mechanisms against cataracts have not been fully explored yet.

Herbal medicines consist of multiple active ingredients, which result in complicated multitarget and multipathway characteristics when acting on diseases. In recent years, a novel TCM network pharmacology research strategy has been widely applied, on the basis of systematic concepts, to the discovery of the underlying mechanism of TCM or herbal medicines against diseases. Like other computational methods [14–16], network pharmacology is a well-established computational methodological theory to reveal the pharmacological mechanism of TCM or herbal medicines. For cataracts, network pharmacology was used to explore the molecular mechanism of various medicines in the treatment of diabetic cataracts, including protocatechualdehyde [17] and *Buddlejae Flos* [18]. As a traditional Chinese herbal medicine, the mechanism of CS in the treatment of cataracts is well suited to be studied using a network pharmacology approach.

In this study, we aimed to systematically elucidate the pharmacological mechanisms of CS against cataracts based on a network pharmacology approach. Firstly, we screened for active ingredients of CS and obtained the targets of the active ingredients. The cataract-related targets were identified through three databases. PPI data were obtained and used to construct a protein-protein interaction (PPI) network, and GO and KEGG enrichment analyses were carried out to find the potential mechanism of CS against cataracts. Molecular docking was carried out to explore the binding affinity of the proteins and compounds. The effects of CS on human lens epithelial cells were also investigated. This study has previously been published as a preprint [19].

## 2. Material and Methods

### 2.1. Data Preparation

#### 2.1.1. Active Compounds and Their Targets in CS

The active compounds in CS were identified and obtained from the Traditional Chinese Medicine Systems Pharmacology Database (TCMSP) (https://tcmspw.com/tcmsp.php) [20]. It gathered the information on herbs, compounds, compound-targets, compound-related diseases, and pharmacokinetic properties of each compound. In this study, the compounds with OB ≥ 30% and DL ≥ 0.18 were identified as active ingredients. The adopted threshold values for OB and DL indicated good oral absorption and suitable characteristics for the drug development of the compounds [20, 21]. In addition, to identify the corresponding targets of CS active compounds, the TCMSP database, STITCH (http://stitch.embl.de/), and the DrugBank database (https://www.drugbank.ca/) were used to find potential targets. Eventually, 13 active compounds of CS were obtained, with a total of 105 targets after removing duplicates.

#### 2.1.2. Potential Target Genes of Cataracts

The cataract-related targets were identified from three public databases, including the GeneCards (https://www.genecards.org/) database, Online Mendelian Inheritance in Man (OMIM, https://www.omim.org/) database, and the MalaCards (http://www.malacards.org/pages/info) database [22–24]. Then, we obtained the standard gene names of the identified targets from the UniProtKB (https://www.uniprot.org/help/uniprotkb/) database.

#### 2.1.3. Construction of the PPI Network

We obtained the PPI data using the plugin Bisogenet [25] of Cytoscape 3.5.1 software, which collected PPI data from six databases, including the Database of Interacting Proteins (DIP™), Biological General Repository for Interaction Datasets (BioGRID), Human Protein Reference Database (HPRD), IntAct Molecular Interaction Database (IntAct), Molecular INTeraction database (MINT), and Biomolecular Interaction Network Database (BIND), and visualized the PPI network of compound targets and disease targets with Cytoscape software.

### 2.2. Network Construction and Analysis

Network analysis can scientifically interpret the complex relationships among herbs, compounds, diseases, and genes [26, 27]. In the study, the compound-target network and the PPI networks of CS compound targets and cataract-related targets were generated by Cytoscape (version 3.7.1) [28]. The MCODE Cytoscape plugin was used to carry out module analysis. The key targets and the central network were screened using a topological method, which adopts six topological parameters, including degree centrality (DC), closeness centrality (CC), betweenness centrality (BC), eigenvector centrality (EC), local average connectivity-based method (LAC), and network centrality (NC), to assess the central attributes of all nodes in a network with the Cytoscape plugin CytoNCA. Specifically, nodes whose values are greater than the mean value for all six parameters were identified as key targets, and the central network composed of these key nodes and the edges between them was also depicted using Cytoscape software.

### 2.3. Enrichment Analysis

In this study, we used online tools of the Database for Annotation, Visualization and Integrated Discovery (DAVID, https://david.ncifcrf.gov, v6.8) to perform the Gene Ontology (GO) and Kyoto Encyclopedia of Genes and Genomes (KEGG) enrichment analysis [29]. Functional categories and pathways with significant changes of *p* < 0.05 were identified. The top 10 GO functional categories and the top 20 pathway categories were used for plotting.

### 2.4. Plant Material and Extraction

Cassiae semen was purchased from a drugstore in Shangyu City, China. The Cassiae semen extract was obtained according to She et al. [30]. Briefly, CS was powdered and then extracted with 70% aqueous ethyl alcohol twice. The extracts were boiled for 1.5 h, and the supernatants were collected and evaporated to dryness under reduced pressure. The dried ethyl alcohol extract of Cassiae semen (EECS) was dissolved in DMSO.

### 2.5. Cell Culture

The human lens epithelial SRA01/04 cell line was purchased from the ATCC (Manassas, USA) and cultured in Dulbecco's modified Eagle's medium (DMEM) containing 10% fetal bovine serum and 1% penicillin/streptomycin at 37°C. To examine the effect of CS on the apoptosis of human lens epithelial cells (HLEC), cells were divided into 3 groups (control, model, and EECS). Cells in the control group were cultured in Dulbecco's modified Eagle's medium (DMEM) containing 10% fetal bovine serum and 1% penicillin/streptomycin. Cells were cultured for 24 h with (EECS group) or without (model group) 2 mg/mL EECS and then exposed to 0.25 mW/cm^2^ UVB. After irradiation, cells were once again cultured in the medium with (EECS group) or without (model group) 2 mg/mL EECS for an additional 6 h. Finally, the cells in three groups were harvested for qPCR and flow cytometry assays.

### 2.6. UVB Irradiation

The apoptosis model of HLEC was established using UVB irradiation. We used a UVB source with a peak spectral emission at 312 nm. It has three fluorescent light tubes (Philips TL 20 W/12 R), and the lights below 295 nm were filtered through a cellulose acetate sheet. Prior to irradiation, cells (80-90% confluence) were washed twice with PBS and supplied with cold PBS. Cells were put on ice and exposed to 0.25 mW/cm^2^ UVB irradiation for 4 min. After exposure, the cells were cultured further for 6 h in a complete medium.

### 2.7. qRT-PCR

Total RNA was extracted using the TRIzol Reagent (Takara Bio, Dalian, China) according to the manufacturer's instructions. Then, total RNA was reverse transcribed into cDNA using PrimeScript RT-polymerase (Takara Bio). RT-PCR reaction was performed with *β*-actin as an internal control in a model 7000 Sequence Detection System (Applied Biosystems, Foster City, CA, USA). The sequences of primers are listed in Table 1. Comparative quantification of genes was determined using the 2^−∆∆Ct^ method.

### 2.8. Cell Apoptotic Rate Assay

AnnexinV-FITC/propidium iodide (PI) staining (Tiangen Biotech, Beijing, China) was used to quantify the amount of cell apoptosis. Briefly, SRA01/04 cells from the control, model, and EECS group were collected and stained with AnnexinV-FITC/PI in a binding buffer for 20 min. The stained cells were then analyzed using the Beckman FC500 MCL flow cytometry system.

### 2.9. Molecular Docking

Molecular docking was performed using CB-Dock (http://cao.labshare.cn/cb-dock/) online tools to predict the binding activities of proteins to compounds and calculate the center and size of the cavity [31]. The PDB formats of proteins were obtained from the RCSB PDB database (http://www.rcsb.org), and the ligand file in SDF formats was derived from the PubChem database (https://pubchem.ncbi.nlm.nih.gov/) [32]. Fulvestrant, (-)-kusunokinin, *α*-bisabolol, SB203580, and HDM201 were selected as inhibitors of ESR1, AKR1B1, CASP3, MAPK14, and TP53, respectively. The combinations of the best docking scores were visualized by PyMOL.

### 2.10. Statistical Analysis

The results are presented as mean ± standard deviation (SD). Differences between groups were assessed by one-way analysis of variance (ANOVA). Calculations were performed using SPSS for the Windows version 13.0 statistical package (SPSS, Chicago, IL). *p* values less than 0.05 were considered statistically significant.

## 3. Results

In our study, a total of 13 active compounds in CS were identified using the ADME model, including rhein, toralactone, stigmasterol, aloe emodin, campesterol, rubrofusarin gentiobioside, rubrofusarin, aurantio-obtusin, obtusin, gluco-obtusifolin, 9,10-dihydroxy-7-methoxy-3-methylene-4H-benzo[g]isochromen-1-one, quinizarin, and CLR. Detailed information is presented in Table 2. Among these compounds, we failed to get target information for rubrofusarin in public databases.

### 3.1. CS Compound-Target Network

**Figure 1:**
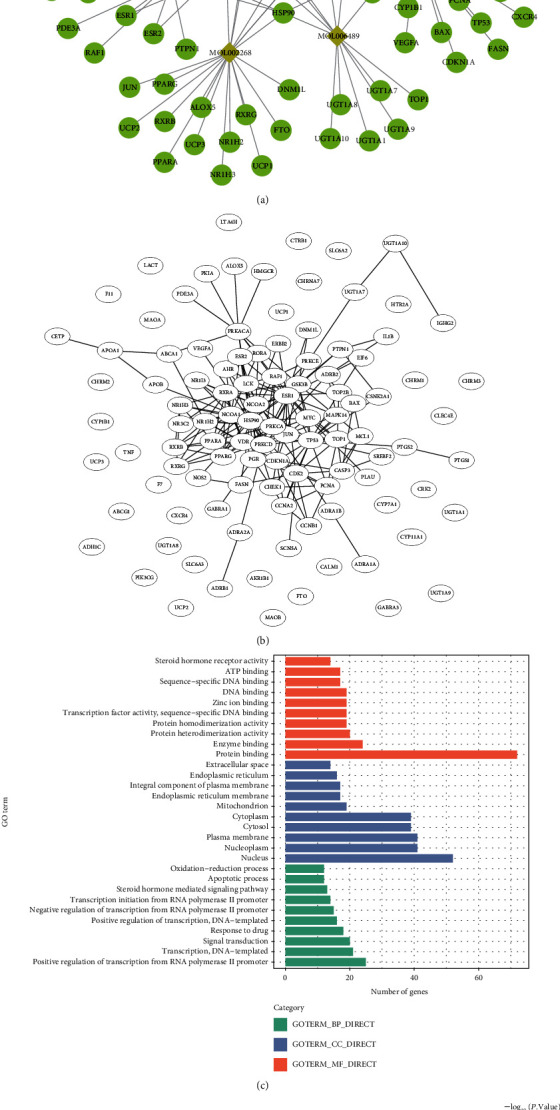
The characteristics of active compounds in CS and their targets: (a) the network of active compounds and their targets; (b) the PPI network of active compounds' targets; (c) top 10 enriched GO terms of compounds' targets; (d) the top 20 enriched pathways of compounds' targets.

The compound-target network consisted of 117 nodes (12 active compounds and 105 targets) and 152 edges, as shown in Figure 1(a). The top 3 compounds in the network with more targets were MOL000471 (aloe emodin, degree = 32), MOL000449 (stigmasterol, degree = 31), and MOL002268 (rhein, degree = 20), indicating their important role in treating cataracts. Furthermore, it showed that many targets were connected and affected by multiple compounds. Prostaglandin-endoperoxide synthase 2 (PTGS2), nuclear receptor coactivator 2 (NCOA2), and prostaglandin-endoperoxide synthase 1 (PTGS1) were the top three targets with a higher number of connected compounds. The PPI network of the compound targets is depicted in Figure 1(b), and the characteristics of CS targets were clarified by GO analysis and KEGG pathway analysis. It revealed that the majority of the potential targets existed in the nucleus with the function of protein binding and were highly enriched in the regulation of transcription, signal transduction, response to drug, apoptotic process, and oxidation-reduction process (Figure 1(c)). In addition, ninety-five significantly enriched pathways (*p* < 0.05) were identified, and the top 20 pathways mainly contained cancer-related pathways, signal transduction pathways, and virus-related pathways (Figure 1(d)).

### 3.2. Cataract-Related Target Genes

**Figure 2:**
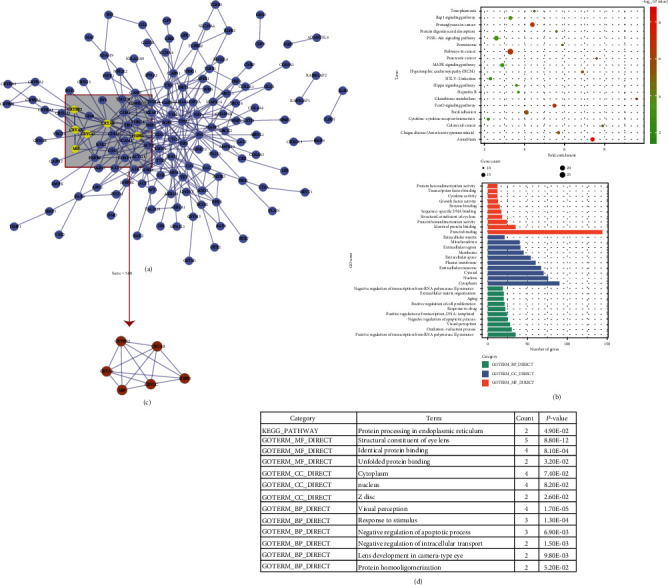
The characteristics of cataract-related targets: (a) the PPI network of the cataract-related targets; (b) KEGG and GO analysis of the cataract-related targets; (c) a subnetwork from module analysis with score = 5.60; (d) GO and KEGG results of the subnetwork from module analysis.

A total of 238 target genes related to cataracts were identified from the OMIM (48), MalaCards (8), and GeneCards (232), after removing the duplicates. The PPI network (removing nodes without any connection) of these targets was constructed (Figure 2(a)), which included 148 nodes and 290 edges. The data of GO analysis and KEGG pathway analysis are shown in Figure 2(b). It revealed that 373 GO terms were significantly enriched (*p* < 0.05), with 281 in the biological process, 43 in the cellular component, and 49 in the molecular function. In addition, a total of 67 pathways (*p* < 0.05) were affected by cataracts, and the top 20 enriched pathways are shown in Figure 2(b), mainly including cancer-related pathways, signal transduction pathways, and virus-related pathways.

In addition, module analysis obtained a cluster of 6 targets with score = 5.60 from the PPI network of cataract target genes (Figure 2(c)). Enrichment analysis showed that these targets were enriched in protein processing in the endoplasmic reticulum and involved in visual perception and response to stimulus (Figure 2(d)), indicating the important role of this cluster in the pathogenesis of cataracts.

### 3.3. CS Anticataract Target Analysis

**Figure 3:**
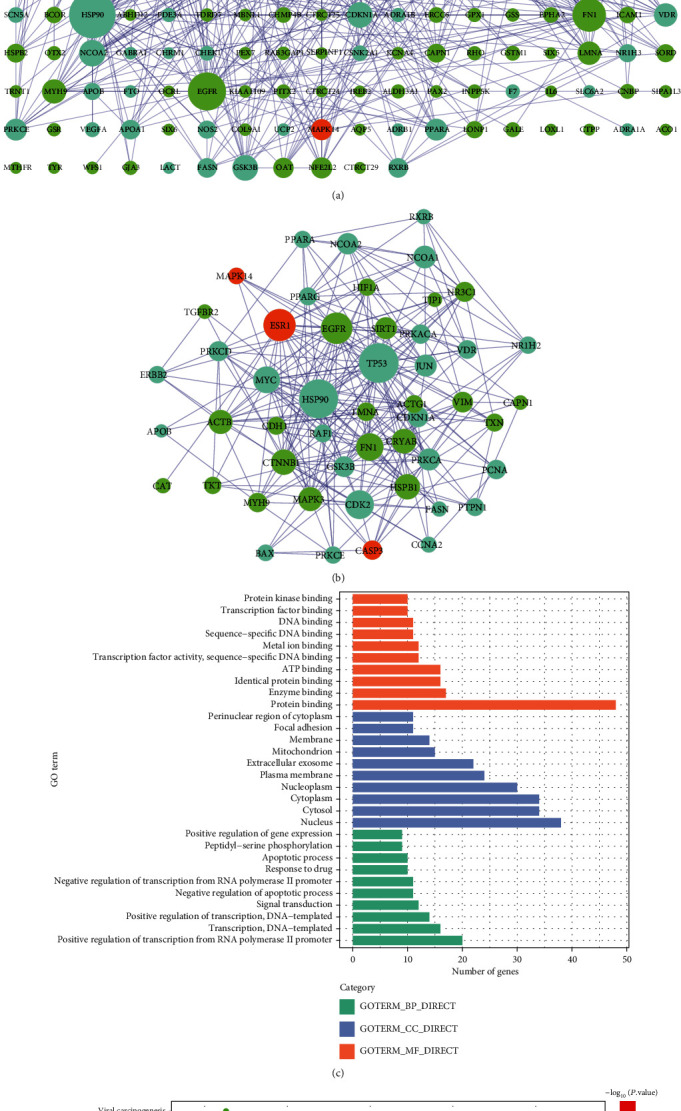
The central network analysis and bioinformatic analysis: (a) the merged PPI network of compound targets and cataract-related targets; (b) central network obtained from the merged network; (c) top 10 enriched GO terms of the key targets from the central network; (d) the top 20 enriched pathways of the key targets from the central network. In (a) and (b), green circles represented compound targets, cyan circles represented disease targets, and orange circles represented shared targets.

We generated the PPI network of potential anticataract targets of CS, as shown in Figure 3(a). It consisted of 335 nodes and 704 edges, and fifty key targets with 251 interactions were screened from the network (Figure 3(b)). In addition, GO analysis showed that two hundred and seventy-nine GO terms were significantly enriched, and the top 10 terms are shown in (Figure 3(c)). These results indicated that various biological processes were involved in the anticataract effects of CS. Moreover, we identified 87 significantly enriched pathways in total, and the top 20 pathways are shown in Figure 3(d).

### 3.4. Compound-Target Docking

**Figure 4:**
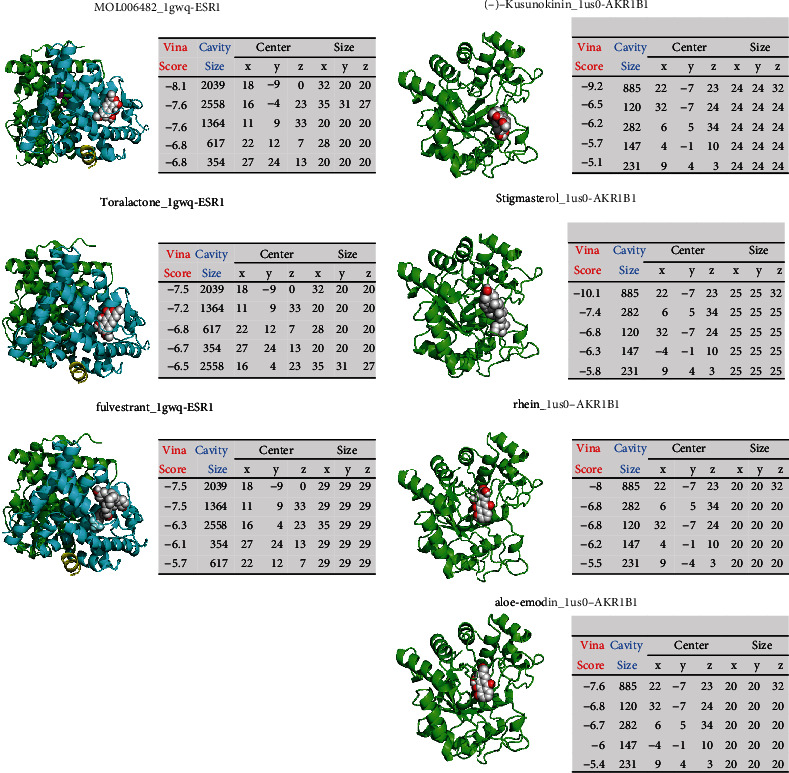
Molecular docking results of the proteins and compounds or inhibitors (1). A sphere and a cartoon chain represent a ligand and a protein, respectively.

**Figure 5:**
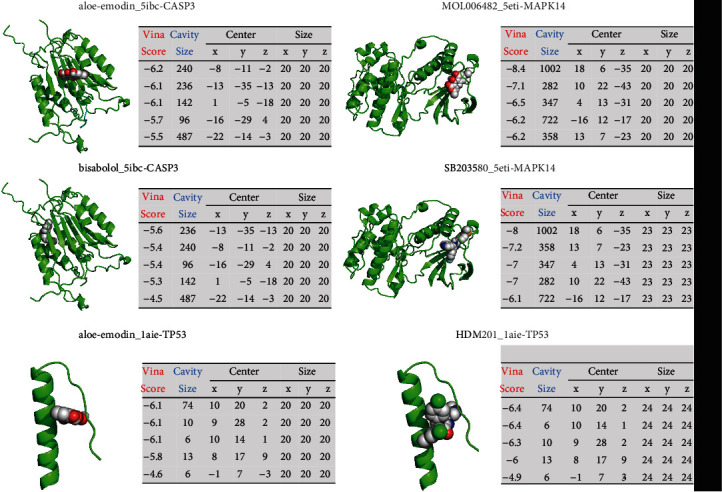
Molecular docking results of the proteins and compounds or inhibitors (2). A sphere and a cartoon chain represent a ligand and a protein, respectively.

Five important targets (ARK1B1, ESR1, TP53, MAPK14, and CASP3) from the network pharmacology analysis were selected to perform molecular docking analysis with their target compounds. Fulvestrant, (-)-kusunokinin, *α*-bisabolol, SB203580, and HDM201 were selected as inhibitors of ESR1, AKR1B1, CASP3, MAPK14, and TP53, respectively. The top 5 cavity sizes and Vina scores of each compound-target or inhibitor-target docking were obtained from CB-Dock. It is generally believed that the lower Vina score indicates a more stable binding state between a protein and a compound. In addition, if a cavity size is close to or bigger than the ligand, the accuracy of docking tends to increase [31]. Molecular docking results showed that the compounds of CS had good binding activities to important targets and were close to or higher than the Vina scores and cavities' size of the protein inhibitors (Figures 4 and 5).

### 3.5. The Effects of CS on HLEC

**Figure 6:**
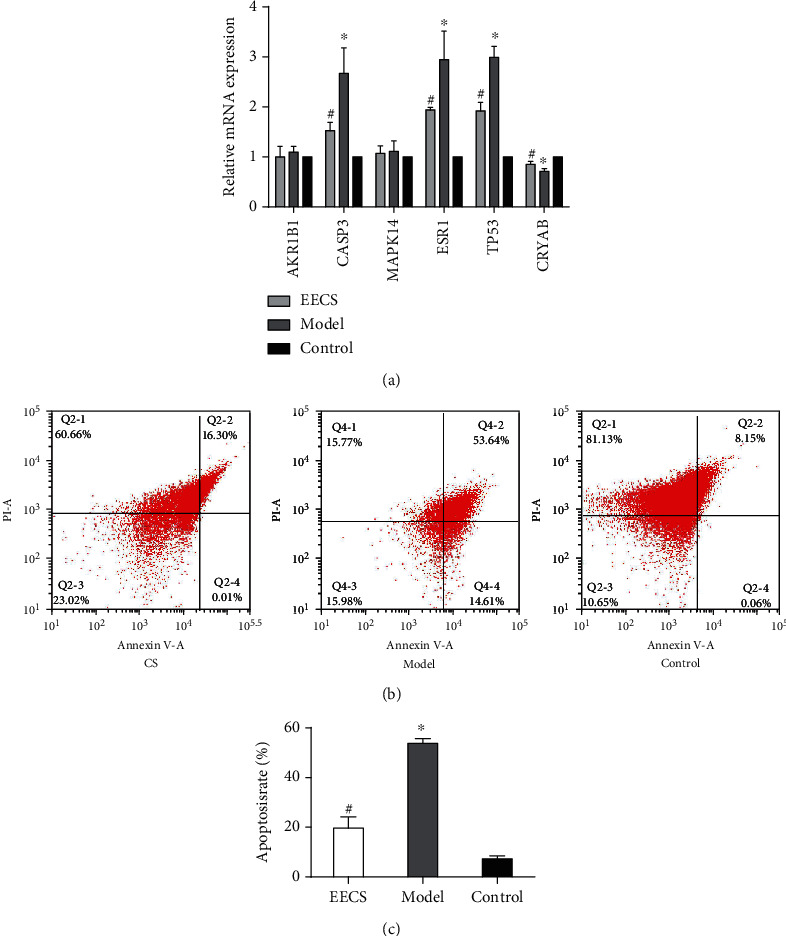
The effect of CS on the gene expression and apoptosis of human lens epithelial cells. (a) The effect of CS on the mRNA expression of AKR1B1, CASP3, MAPK14, ESR1, TP53, and CRYAB. (b) The effects of CS on the apoptosis of human lens epithelial cells. The groups were identified as follows: (i) CS: apoptosis cell model of human lens epithelial cells treated with CS, (ii) model: apoptosis cell model of human lens epithelial cells, and (iii) control: human lens epithelial cells. ^∗^*p* < 0.05 compared to the control group, ^#^*p* < 0.05 compared to the model group.

Excessive apoptosis of lens epithelial cells is implicated in the pathogenesis of several types of cataract formation. Herein, we detected the effect of CS on the mRNA expression of several hub genes located in the central network and the apoptosis of HLEC. The results demonstrated that UVB induced the upregulation of CASP3, TP53, CRYAB, and ESR1 (Figure 6(a)) and HLEC apoptosis (Figures 6(b) and 6(c)). Meanwhile, CS treatment could not only restore the dysregulated expression of CASP3, TP53, CRYAB, and ESR1 in HLEC but also prevented the HLEC apoptosis induced by UVB (Figure 6). These data indicated that CS may treat the cataract by inhibiting the apoptosis of lens epithelial cells.

## 4. Discussion

Cataracts, the major cause of blindness, are characterized by blurry vision. It has been reported to be associated with various risk factors, including smoking, hypertension, steroid consumption, diabetes, and ionizing radiation [33, 34]. CS is a classical herb used to remove “liver fire” for improving eyesight. It has been clinically used to treat ophthalmic diseases, such as cataracts, myopia, and dry eye symptoms, for thousands of years in China. In this study, a network pharmacology approach was applied to comprehensively elucidate potential mechanisms of the beneficial effects of CS on cataracts.

In this study, we identified 13 active compounds in CS and 105 potential targets of these active compounds in total, and 238 cataract-related targets were also obtained from the three public databases. Four genes, including ESR1, MAPK14, CASP3, and AKR1B1, were shared between CS compound targets and cataracts' targets, indicating their possible anticataract action. Central network analysis obtained a central network with 50 key targets, which significantly enriched the pathways correlated with cataracts, such as the thyroid hormone signaling pathway, PI3K-Akt signaling pathway, and MAPK signaling pathway. The potential mechanisms of CS against cataracts were for the first time comprehensively investigated in the present study, which laid a theoretical foundation for the clinical application of CS in the treatment of cataracts and further research.

Among the active compounds in CS, the top three active ingredients with the most targets were aloe emodin, stigmasterol, and rhein, indicating their potential role in the treatment of cataracts. Aloe emodin is an anthraquinone derivative, which possesses the antiangiogenic effect on laser-induced choroidal neovascularization by inhibiting the HIF-1*α*/VEGF signaling pathway and has the potential to be developed for the prevention and treatment of diabetic retinopathy [35]. In addition, aloe emodin metabolites could regulate cell energy, antioxidation, and the phosphorylation of ERK kinases to decrease NMDA-induced apoptosis of retina ganglion cells [36]. Stigmasterol is steroid alcohol with immune-modulatory properties either alone or as a component of phytosterol mixtures [37]. It was reported to attenuate both innate and adaptive immune responses and inhibit inflammatory cell recruitment and oxidative stress as well [12, 38]. Rhein is a major component of many medicinal herbs with various properties, including anti-inflammatory, antioxidant, and anticancer activities [39–41]. Oxidative stress has been observed in the onset and progression of cataractogenesis [42, 43], and antioxidants and free radical scavengers have been suggested as potential drugs for the management of cataracts. Hence, the therapeutic effect of CS on cataracts may, at least in part, result from the antioxidant activity of compounds.

Network analysis suggested that four shared targets may play crucial roles in the treatment of cataracts, including aldose reductase (AKR1B1), caspase-3 (CASP3), mitogen-activated protein kinase 14 (MAPK14), and estrogen receptor (ESR1). AKR1B1, an NADPH-dependent aldo-keto reductase, is involved in diabetic cataracts and retinopathy [44]. A previous study reported that elevated AKR1B1 can increase AcSOD2 and RAGE-induced epithelial-mesenchymal transition (EMT) in the epithelial human lens of DM cataracts via decreasing AMPK activation [45], and the significance of AKR1B1 in the mediation of sugar-induced lens opacification has also been confirmed [46], indicating the potential use of AKR1B1 inhibitors in preventing cataractogenesis. CASP3 is one of the central mediators of apoptosis and has been revealed to be associated with the pathogenesis of cataracts [47]. MAPK14 plays an important role in cataract formation, owing to the activation of MAPK14 which can lead to the induction of cataracts [48]. Estrogen therapies showed protection against age-related cataracts in humans and rodent models, and ER*α* overexpression has previously been reported in lens epithelial cells [49], indicating that estrogen protection may result from direct interactions with its receptors in the eye. In addition, TP53 with the highest degree in the central network indicated its important role in the treatment of cataracts, and previous studies also confirmed that p53 is involved in the pathogenesis of cataracts and mediates the anticataract effect of certain compounds [50]. Module analysis and central network analysis revealed that *α*B-crystallin (CRYAB) may play an important role in the treatment of cataracts. It is a chaperone that maintains protein stability and preserves lens transparency [51, 52] by preventing proteins from aggregating via low-affinity amphipathic interactions [53]. The docking results demonstrated that the compounds exhibited good affinity to these critical targets.

As demonstrated in network pharmacology analysis, the hub genes were enriched in the apoptosis process. Meanwhile, the apoptosis of lens epithelial cells contributes to cataract development. Therefore, we investigated the impacts of CS on the apoptosis of human lens epithelial cells. As expected, CS treatment could reduce the UVB-induced elevated apoptosis rate of HLECs. Several apoptosis-related genes were also regulated by CS, including CASP-3, TP53, ESR1, and CRYAB, indicating that CS may prevent HLEC apoptosis via regulating these hub genes. Although the gene expression of MAPK14 and AKR1B1 was not affected by CS treatment, the activities of these proteins required further validation to identify their roles in the treatment of cataracts by CS.

In addition, the PPI data of compound targets and cataract-related targets were obtained to construct the PPI network. Enrichment analysis of these two sets of targets revealed a series of shared pathways, such as the PI3K-Akt signaling pathway, MAPK signaling pathway, and FoxO signal pathway. To obtain the central network of CS anticataract targets, we merged the PPI network of compound target and cataract-related targets. KEGG pathway enrichment analysis showed that the key targets of CS against cataracts were mainly enriched in the thyroid hormone signaling pathway, MAPK signaling pathway, and PI3K-Akt signaling pathway, indicating the involvement of these pathways in the treatment of cataracts.

The thyroid hormone signaling pathway participates in the regulation of growth, development, and glucose metabolism. The modulation of glycolysis and carbon flux reprogramming can increase the glutathione (GSH) syntheses and activate the antioxidant enzymes [54], which are beneficial for protecting the lens from oxidative stress leading to opacification. A previous study has reported a decrease in lenticular GSH levels that occurred during the formation of most cataracts [55]. As a substrate for glutathione peroxidase, GSH can destroy lipid peroxide (LPO) and hydrogen peroxide, which mediate the hepatic oxidative stress and contribute to cataract formation [56]. Thence, a possible GSH-consuming factor is considered to be cataractogenic. It was believed that the stimulated glycolysis results in the restoration of hepatic ATP by recovering the citric acid cycle, consequently facilitating *de novo* synthesis of GSH. However, Kosano et al. demonstrated that thyroxine treatment accelerated the GSH-GSSG cycle rather than *de novo* synthesis of GSH to maintain a certain level of hepatic GSH necessary for reducing elevated LPO [57].

The MAPK signaling pathway is another enriched pathway for CS in the treatment of cataracts, which involves various cellular functions, including cell proliferation, differentiation, and migration. Hashida et al. found the association of cataract formation with the upregulation of MAPK cascade protein [58]. In addition, the MAPK/ERK1/2 signaling pathway also participates in the regulation of human lens epithelial cells' function by the *γ*-Klotho gene [59]. Andrographolide is confirmed to be useful in curbing EMT-mediated posterior capsular opacification because it helps maintain epithelial characteristics by regulating EMT markers and inhibiting the MAPK signaling pathway in lens epithelial cells (LECs) [60]. Peng et al. demonstrated that p-coumaric acid acts as a potential therapeutic drug for cataracts by suppressing the apoptosis of human LECs via modulating the MAPK signaling pathway [61]. Therefore, the role of the MAPK signaling pathway for CS against cataracts should also be validated in the future.

Notably, the PI3K-Akt signaling pathway might be associated with the ingredients of CS and anticataract activity. It has been demonstrated that the PI3K-Akt signaling pathway is involved in the pathogenesis of cataracts [62, 63]. Meanwhile, a series of compounds exhibited an effect on cataracts by modulating the PI3K-Akt signaling pathway, such as alkylphosphocholine erufosine [64], quercetin [65], and andrographolide [66]. Many of the active ingredients in CS have been proven to regulate the PI3K-Akt signaling pathway, including rhein [13], aloe emodin [67], and rubrofusarin [68], indicating that CS acted on cataracts possibly through the PI3K-Akt signaling pathway.

## 5. Conclusion

In conclusion, this study used a network pharmacology approach to explore the potential mechanisms of CS acting on cataracts. Key targets and pathways involved in the treatment of cataracts using CS were identified, which provided evidence for the clinical application of CS in cataract treatment and further studies. CS treatment regulated the gene expression of several hub genes in HLEC and prevented the apoptosis of HLEC, which may contribute to the cataract treatment. However, from a critical point of view, further experiments are required to validate other findings. This study also provided clues to evaluate the synergy of herbs in the treatment of other complex diseases.

## Figures and Tables

**Table 1:** Summary of PCR primer sequences used for RT-PCR.

Gene name	Primer sequences (5′-3′)
AKR1B1	F: TTTTCCCATTGGATGAGTCGG R: CCTGGAGATGGTTGAAGTTGG
Caspase-3	F: TGGAACAAATGGACCTGTTGACC R: AGGACTCAAATTCTGTTGCCACC
MAPK14	F: GGGGCAGATCTGAACAACAT R: GAGCCAGTCCAAAATCCAGA
ESR1	F: AGGCTTTGTGGATTTGAC R: CCAAGAGCAAGTTAGGAG
TP53	F: ACCCAGGTCCAGATGAAG R: CACTCGGATAAGATGCTGA
CRYAB	F: CTT TGA CCA GTT CTT CGG AG R: CCT CAA TCA CAT CTC CCA AC
*β*-Actin	F: AAG TAC TCC GTG TGG AT C GG R: ATG CTA TCA CCT CCC CTG TG

F: forward; R: reverse.

**Table 2:** Active ingredients of Cassiae semen.

MOL ID	MOL name	2D structure	OB (%)	DL
MOL002268	Rhein	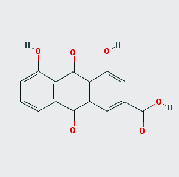	47.07	0.28
MOL002281	Toralactone	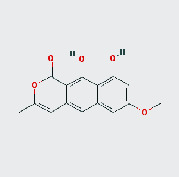	46.46	0.24
MOL000449	Stigmasterol	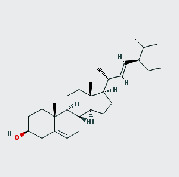	43.83	0.76
MOL000471	Aloe emodin	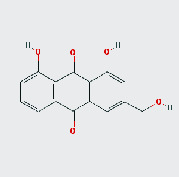	83.38	0.24
MOL005043	Campesterol	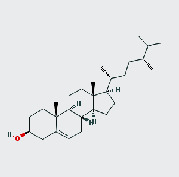	37.58	0.71
MOL006465	Rubrofusarin gentiobioside	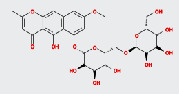	40.12	0.67
MOL006466	Rubrofusarin	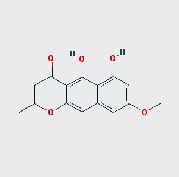	45.55	0.24
MOL006472	Aurantio-obtusin	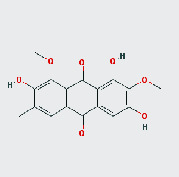	31.55	0.37
MOL006475	Obtusin	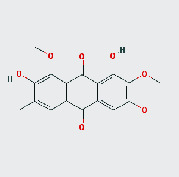	81.43	0.4
MOL006481	Gluco-obtusifolin	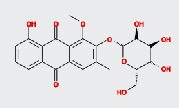	42.41	0.81
MOL006482	9,10-Dihydroxy-7-methoxy-3-methylene-4H-benzo[g]isochromen-1-one	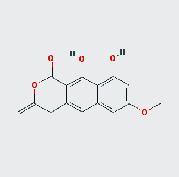	63.25	0.24
MOL006489	Quinizarin	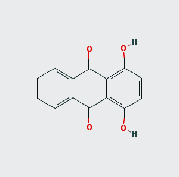	47.34	0.19
MOL000953	CLR	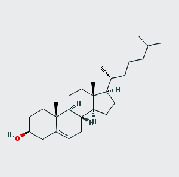	37.87	0.68

## Data Availability

The datasets of CS targets and cataract-related targets used and/or analyzed during the current study are available from the corresponding author on reasonable request.

## Abbreviations

CS: Cassiae semen
PPI: Protein-protein interaction
OB: Bioavailability
DL: Drug-likeness
TCMSP: Traditional Chinese medicine systems pharmacology database
DIP™: Database of Interacting Proteins
BioGRID: Biological General Repository for Interaction Datasets
HPRD: Human Protein Reference Database
IntAct: IntAct Molecular Interaction Database
MINT: Molecular INTeraction database
BIND: Biomolecular Interaction Network Database
DC: Degree centrality
CC: Closeness centrality
BC: Betweenness centrality
EC: Eigenvector centrality
LAC: Local average connectivity-based method
NC: Network centrality
GO: Gene Ontology
KEGG: Kyoto Encyclopedia of Genes and Genomes
PTGS2: Prostaglandin-endoperoxide synthase 2
NCOA2: Nuclear receptor coactivator 2
PTGS1: Prostaglandin-endoperoxide synthase 1
AKR1B1: Aldose reductase
CASP3: Caspase-3
MAPK14: Mitogen-activated protein kinase 14
ESR1: Estrogen receptor
CRYAB: *α*B-Crystallin
GSH: Glutathione
LPO: Lipid peroxide
LECs: Lens epithelial cells.
